# Tools to assess employment readiness for colorectal cancer survivors: A scoping review

**DOI:** 10.1002/cam4.6432

**Published:** 2023-08-09

**Authors:** Mingshuang Ding, Elise Gane, Harry Wiffen, Venerina Johnston

**Affiliations:** ^1^ School of Health and Rehabilitation Sciences The University of Queensland St Lucia Queensland Australia; ^2^ EBSCO Information Services Brisbane Queensland Australia; ^3^ Physiotherapy Department Princess Alexandra Hospital Ipswich Massachusetts USA; ^4^ Royal Brisbane and Women's Hospital Brisbane Queensland Australia; ^5^ School of Health and Medical Sciences, Faculty of Health, Engineering and Sciences University of Southern Queensland Darling Heights Queensland Australia

**Keywords:** cancer, colorectal, employment, measurement, readiness, survivor

## Abstract

**Background:**

The ability to return to work and remain at work is an important recovery milestone after a cancer diagnosis. With the projected number of colorectal cancer patients of working age likely to increase, it is important to identify when a person is ready to resume work. There are many employment‐related tools available to help people return to work after injury or illness; however, it is unknown which may be suitable for a person with colorectal cancer.

**Aim:**

To identify tools related to employment readiness in colorectal cancer survivors and to chart the relevant factors of employment assessed by these tools.

**Method:**

Literature searches were performed in PubMed, CINAHL, Embase and Medline, the Cochrane library and PsycINFO using search terms around cancer, survivorship and employment to identify all peer‐reviewed articles published in English up to June 2022.

**Results:**

Thirty‐five studies used a total of 77 tools focused on assessing employment issues experienced by people with cancer in general. Four tools were used with colorectal cancer survivors. None considered all relevant employment‐related factors for colorectal cancer survivors.

**Conclusion:**

Tools used to identify return‐to‐work and remain‐at‐work were not specific to colorectal cancer. There are a range of existing tools that collate some, but not all, of the domains and outcome criteria required to meet the employment needs of colorectal cancer survivors. To optimize work outcomes for the working colorectal cancer population, a specified tool is warranted.

## 1 BACKGROUND

In 2021, it was estimated that 150,782 new cases of cancer were diagnosed in Australia.^1^ Early detection and more efficacious treatments have seen increasing survival rates following a cancer diagnosis. Subsequently, a growing number of cancer survivors are seeking to return to meaningful life participation that may have been unavailable to them during treatment. A particularly relevant area of daily living that cancer survivors are often excluded from, due to the effects of both their illness and treatment, is employment.^2^ In general, cancer has a negative impact on employment, with studies estimating that between 10% and 38% of people working when diagnosed do not return to work following cancer treatment.^3^, ^4^, ^5^

For many cancer survivors, work is a vital aspect of re‐establishing normality. In wider society, work serves a range of functions beyond that of material well‐being. In studies of people with cancer, work is perceived as a means of reducing or avoiding social isolation, boredom, loss of self‐esteem, financial hardship, and a way of enabling people to regain a sense of normality, and a positive concept of self and identity.^6^, ^7^ Several studies have demonstrated that reduced capacity to return to, or engage fully in work was associated with poorer quality of life outcomes.^8^, ^9^, ^10^ Cancer survivors have reported significant psychological and physical stressors in instances where they have returned to their previous roles before being ready.^11^, ^12^ The decision of determining if, and when, a person with cancer is ready to return to work can be challenging due to the ongoing nature of treatment, such as extensive follow‐up and screening, as well as treatment‐related side effects. For example, cancer‐related fatigue is the most frequently reported issue preventing cancer survivors from returning to work.^11^, ^13^ In addition, medical interventions for the treatment of cancer (surgery or chemotherapy) can have persisting and unexpected impact on an individual's physical and cognitive function, negatively influencing productivity at work. Those that do return with a higher degree of comorbidity have been shown to have greater difficulty re‐integrating into the work environment.^14^ Thus, there are many factors that influence the return‐to‐work process which need to be considered and addressed to inform employment readiness after a cancer diagnosis.

One common cancer with improving prognosis is colorectal cancer.^1^, ^15^, ^16^ It is the third most commonly diagnosed cancer worldwide, with the highest incidence among developed countries.^17^, ^18^ However, the introduction of the biennial faecal occult blood test screening programme has seen the prognosis of colorectal cancer steadily improving via early detection, with 5‐year survival rates reaching 70% in Australia.^1^ The median age for diagnosis for colorectal cancer is 70 years in Australia, indicating that approximately 50% of colorectal cancer patients are still of working age. With the age‐related pension threshold set at 65.5 years and set to increase, it is expected that the number of colorectal cancer patients of working age will significantly increase in the future. Survivors of colorectal cancer have reported unique and challenging experiences after their treatment and when returning to work.^19^ This is in part due to their post‐treatment bowel function and the psychological effects and stigma associated with having a stoma.^19^, ^20^ These procedures are definitive in up to 22% of cases.^21^ Little is known about the process of returning to work after colorectal cancer and specifically how to support clinicians and individuals in identifying readiness to return to work.

Assessing the return‐to‐work process is difficult as it is highly individualized and involves numerous stakeholders such as employers and healthcare professionals.^22^ There is extensive research investigating the return‐to‐work process for people with compensable musculoskeletal conditions and mental health conditions but limited research with clinical populations such as cancer.^23^, ^24^, ^25^, ^26^ At present, there are no known tools specific to colorectal cancer and return‐to‐work described in clinical guidelines in Australia or globally.^27^ Recent systematic reviews have not identified tools specific for colorectal cancer and work although this was not their primary aim.^28^ Hence, a scoping review of the literature was conducted to explore the tools available for assessing readiness for work after cancer with particular emphasis on colorectal cancer. The aims of this scoping review are as follows: to identify tools related to employment readiness in colorectal cancer survivors and chart the relevant factors of employment assessed by these tools. This information will be useful for the development of new tools or modification of an existing tool.

## 2 METHODS

### 2.1 Study design

Scoping reviews are useful for a number of academic needs particularly in identifying the types of available evidence in a given field; to clarify key concepts/definitions in the literature; to examine how research is conducted on a certain topic or field; to identify key characteristics or factors related to a concept; as a precursor to a systematic review and to identify and analyse knowledge.^29^ As a result, a scoping review was selected to achieve the aims of this study due to the limited literature in the field. The framework by Arksey and O'Malley^29^ was adopted utilizing the first five of six stages (Stage 6, consultation exercise, is optional): Stage I, identifying the research question; Stage 2, identifying relevant studies; Stage 3, study selection; Stage 4, charting the data; Stage 5, collating, summarizing and reporting the results. This scoping review was prepared according to the PRISMA‐ScR reporting guidelines.^30^

### 2.2 Stage 1: identifying the research questions

The research questions for this review were as follows (1) What tools are available to inform decision‐making about returning to work or remaining at work in colorectal cancer survivors? and (2) What employment‐related factors are assessed by these tools?

### 2.3 Stage 2: identifying relevant studies

The second stage of the scoping review process identified the criteria for inclusion of studies in the review. Although a scoping review is designed to cover a broad spectrum of literature, inclusion and exclusion criteria guided the search and helped to filter the literature. Thus, the scoping review included published peer‐reviewed articles that were retrieved from the following electronic databases: PubMed, CINAHL, Embase, Medline, the Cochrane library and PsycINFO. To capture all relevant published material, no limit was set for the publication date to June 2022. Reference chaining (a review of links found through the electronic search) was undertaken to ensure that all possibly relevant articles could be included in the scoping review. Keywords (including Medical Subject headings [MeSH] and ‘All field’ search terms) used to execute the literature search covered the concepts “cancer,” “survivorship,” “employment,” “sick” and “measurement tool,” joined by the Boolean operator “AND” to produce the search results (Table 1).

**TABLE 1:** Keywords used in the literature search of electronic databases.

Cancer	Survivorship	Employment	Sick	Measurement tool
Cancer Carcinoma Malignancy Neoplasm Oncology Tumour	“after/post cancer” “after/post treatment/s” “cancer survivor/s” Follow‐up “late effects” “delayed effects” “long term effects” “life after cancer” “life after cancer care/treatment” “living with cancer” “long term survivor/s” “survivor need/s” “after/post treatment/s” “after/post‐surgery” “after/post‐chemotherapy” “after/post‐radiotherapy” Survivor/s Survivorship “survivorship care”	Employment “employment ability” “employment loss” Work “work ability” “work disability” “work functioning” “work limitation” Job Productivity “return to work” “return to work” “remain at work” “remain‐at‐work” “stay at work” “stay‐at‐work”	Sick Absence “sick leave” “time‐off” “sickness absence”	“measurement tool” “measurement instrument” Survey Questionnaire Assessment Interview Observation Report

During an initial search, it was discovered that there was a limited body of evidence specific to colorectal cancer, restricting the exploration of potential publications and tools. Therefore, the scope of the review was widened to all cancers. For the purposes of this review, ‘return‐to‐work’ is defined as the process whereby a person with a diagnosis of cancer plans to return to their pre‐diagnosis paid employment or seek a new job after a period of non‐employment in which medical treatment was received.^31^ An academic librarian was consulted and advised on the most appropriate MeSH terms for the search and how to modify MeSH terms for the different databases used. Based on this extensive exploratory scoping phase, the search strings for each database were finalized.

### 2.4 Stage 3: study selection

The results of the searches from different databases were consolidated and duplicates excluded. Three authors (MD, HW and FA) then independently screened the titles and abstracts of the articles against the eligibility criteria. Based on the aims of this review, the following inclusion criteria were applied to search results: peer‐reviewed literature of any design, only full text, no date restriction, included cohorts of people with a cancer diagnosis, and studies that had tools related to employment readiness in colorectal cancer survivors. Only studies in English were included. Data, such as conference abstracts, commentaries, books and book reviews, editorial articles and non‐peer reviewed grey literature, including health service policy and procedure documents were excluded. Also, studies on children and including only non‐working populations were excluded. Disagreements about study eligibility were resolved among the three screening authors (MD, HW and FA) by consensus discussion. Studies that included tools with a few items about employment or reported on incidence/prevalence or measures of employment status or financial burden of cancer on work were excluded as readiness for work is a multifactorial construct.^32^ Hence, only those tools that considered several medical factors, mediator variables and outcome criteria related to employment readiness were included in this review. The quality of each study was rated using the Mixed Methods Appraisal Tool (MMAT).^33^

### 2.5 Stage 4: charting the data

Based on the preliminary scoping phase, a data extraction framework was developed. The framework was pilot tested by two authors (MD and HW) on a sample of the included studies (10% of the complete list of retrieved studies) to ensure that the coding framework was consistently applied.

Two authors (MD and HW) independently extracted the data from each included study. Each included study was assessed to determine the type of cancer that was being examined, and the tools used to determine employment readiness. Discrepancies in extracted data were discussed between the research team until consensus was reached.

### 2.6 Stage 5: collating, summarizing and reporting the results

Analysis and conceptual synthesis of the data collected using the data extraction framework provided information on previous research undertaken around employment readiness for all cancer survivors. It is well understood that a range of variables can impact the transition back to the workplace, long‐term work productivity or job retention among cancer survivors. To evaluate the utility of a tool in assessing readiness for work in someone with cancer, it is valuable to consider which of these contributory factors the tool assesses, as well as its relevance to the outcome of interest. There are several existing models that document the factors that need to be considered for people with cancer wanting to return to work or maintain their employment. The most comprehensive of these include the models developed by Feuerstein et al.,^34^ Mehnert^35^ and Chow et al.^36^ While the terms “model” and “framework” may denote different meanings in certain contexts, the terms are used interchangeably in the papers by Chow et al. and Feuerstein et al., which would suggest that within this context there is no significant practical difference between the two. As such, we have assessed both models and frameworks to develop the analytical framework for our discussion, focusing on the Mehnert model.

**FIGURE 1:**
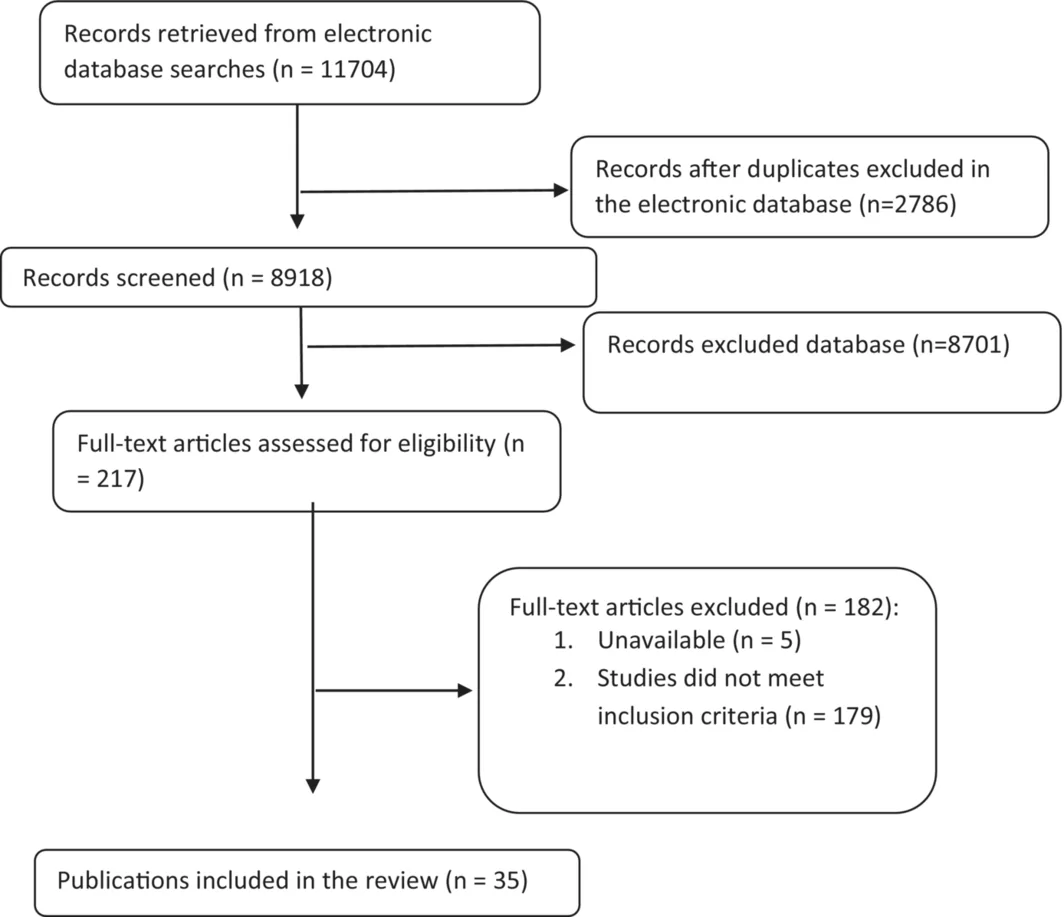
The scoping review selection/screening process.

In 2010, Feuerstein et al. developed the Cancer & Work model, a framework to aid in conceptualizing cancer‐induced employment issues experienced by survivors both in the short and long‐term (Figure 1).^36^, ^37^ The model was designed both as a framework for future research, as well as for clinical and workplace application, such as in guiding the evaluation, prevention and management of survivors who experience problems returning to and/or remaining at work and assisting those with cancer‐related problems to maintain or enhance their abilities at work. Development of the model was based on a systematic search of literature which identified 45 studies on work and cancer. The Cancer & Work model outlined seven broad categories (cancer survivor characteristics, health and well‐being, symptoms, function, work demands, work environment and policies, procedures and economic factors) of variables associated with four work outcomes (return to work, work ability, work performance and sustainability). The model does not indicate the direction of links between policies, procedures and economic factors to other variables in the model for the sake of visual simplicity. However, it is recognized that these factors can influence and be influenced by each of the other categories within the model. Additionally, the model does not presuppose the importance of one outcome over another, rather the outcome/s of interest often depends on the context of the model's use and the motivations of the specific stakeholder implementing the model.

The development of Mehnert's^36^ model for the investigation of work‐related aspects in cancer survivorship was based on the results of a systematic review of 64 studies on return to work and employment in cancer survivors. This model encompasses a range of independent factors, mediating factors and outcome criteria that have guided research about work and employment in cancer survivorship throughout recent years. Specifically, the model outlines independent variables relating to the disease and treatment, as well as six categories of mediating variables (1) demographic factors such as age, gender, education and income; (2) impairments and health‐related factors such as pain, fatigue and physical symptoms; (3) psychosocial factors such as distress, depression, anxiety and the availability of social support; (4) motivational factors such as work satisfaction; (5) work‐related factors related to the type or nature of work, work demands and responsibilities; and ^6^ variables associated with work‐related interventions and care such as vocational training and rehabilitation services. Work‐related outcome criteria consist of a wide range of variables such as continuity of work, employment and return to work, work ability, absence from work and career changes. Further outcome variables pertain to quality of life, social re‐integration and psychological well‐being, as well as economic factors such as the cost of declining work productivity.

Chow et al.^37^ developed their conceptual framework on return to work among cancer survivors based on a systematic review of 27 studies. This framework serves to assist policy makers in addressing issues pertaining to return to work among cancer survivors by highlighting various modifiable factors which represent potential areas of intervention. Specifically, the framework outlines five different categories of factors which impact the primary outcome of return to work. These include environmental factors, personal factors, work demand‐work ability, health status and financial factors. Several sub‐groups of variables are listed within each of these categories. Additionally, arrows provide a visual depiction of the direction of relationships between the factors within the framework.

Many similarities exist between the three models discussed above. Most notably, they are all multivariate in nature, considering the potential impacts of a range of factors related to the individual and their disease, as well as the physical and psychosocial aspects of work. However, several key differences also exist between the three studies and their respective models. Firstly, in terms of the methods and results of the systematic reviews themselves, there were considerably fewer studies included in the review by Chow et al. despite this study being conducted after the publication of the other two papers and including articles published over a broader time period (24 years compared to 10 years in the other two studies). In addition, Chow et al. included both quantitative and qualitative studies (i.e. cancer patients' self‐reported data or point of view on employment‐related issues), whereas Feuerstein et al. and Mehnert only included articles which employed quantitative methodology. It is worth noting that despite being published most recently, the paper by Chow et al. did not reference the studies undertaken by Feuerstein et al. or Mehnert. In fact, Chow et al. stated that at the time of their study, there was no pre‐existing systematic review on the relationship among the various factors associated with return to work or remain in employment.

The three models themselves also differ in terms of their content, structure and intended application or purpose. Mehnert's model was primarily constructed for research purposes, while the other two models were designed for more practical application. The Cancer & Work model was developed for use in both clinical and workplace settings to support cancer survivors in returning to or remaining at work. The conceptual framework constructed by Chow et al. primarily serves as an aid for policy makers in reviewing the current healthcare delivery system by highlighting potential areas of intervention to address issues pertaining to work among cancer survivors. All three models incorporated similar categories of factors which impact work outcomes, including those relating to the individual's health and socioeconomic status, as well as work‐related factors. However, compared to those by Feuerstein et al. and Chow et al., Mehnert's model included a far more detailed outline of these mediating factors, with specific examples of such variables listed under each broad category. Both Feuerstein et al. and Chow et al. utilized unidirectional and bidirectional arrows to depict the complex interplay between the various factors and outcomes in their models. This is lacking in Mehnert's model, which instead merely suggests a one‐way progression from independent and mediator variables to outcomes as a whole.

Finally, the three models include different outcome variables. The conceptual framework by Chow et al. only considered the primary outcome of return to work, while both Feuerstein et al. and Mehnert included several categories of outcome criteria within their models. The Cancer & Work model by Feuerstein et al. focussed solely on various work outcomes, such as return to work, work ability, work performance and sustainability. In comparison, one of the distinguishing features of Mehnert's model is that it considers psychosocial and economic outcomes in addition to work outcomes, and as such is more holistic and comprehensive. Feuerstein et al. addressed long‐term employment outcomes, not just return to work immediately following cancer diagnosis or treatment.

Upon consideration of the strengths and limitations of each of the models Mehnert's model was chosen to guide this scoping review and aid in the identification of tools for determining work readiness in colorectal cancer survivors for several reasons. This model was based on the most extensive review of the literature, with the greatest number of included articles of the three studies. Mehnert's model is highly comprehensive, with specific examples of mediating factors listed within each category of variables. Mehnert's model is the most holistic of the three, as it considers psychosocial and economic outcomes in addition to the work outcomes included in the other two models.

## 3 RESULTS

### 3.1 Description (included and excluded studies)

The flow of search results through the stages of the review is presented in Figure 1. After removing duplicates, 8918 titles and abstracts were screened to identify 217 cancer‐related abstracts. After full‐text screening, 181 articles were excluded, leaving 35 articles eligible for this review.

### 3.2 Characteristics of included papers on cancer and employment

The characteristics of the 35 studies related to cancer and employment included in the final discussion are listed in Table 2. Of these, 17 were cross‐sectional studies,^8^, ^9^, ^14^, ^37^, ^38^, ^39^, ^40^, ^41^, ^42^, ^43^, ^44^, ^45^, ^46^, ^47^, ^48^, ^49^, ^50^ five were prospective cohort studies,^51^, ^52^, ^53^, ^54^, ^55^, ^56^ three were randomized controlled trials (RCTs),^57^, ^58^, ^59^ two used data from national registers,^60^, ^61^ and there was one study each for the following study designs: retrospective study,^62^ exploratory survey,^63^ descriptive correlational survey,^64^ study design report,^10^ longitudinal prospective intervention study^65^ and single arm trial.^66^ The studies were conducted in a variety of global locations, including India,^37^ Japan^40^ and Hong Kong.^38^ The majority however occurred throughout Europe. The studies were published between 2014 and 2022. Studies included participants between the ages of 18 and 90 years. The gender split of the participants varied with studies, ranging from 100% female and male in studies examining breast and prostate cancers, respectively.

**TABLE 2:** Characteristics of included papers on cancer and employment (*N* = 35).

No.	First author, year	Study aim/s	Study design and sample size	Participants (age/mean age, gender, cancer type)	Tool used to measure employment outcomes or factors potentially influencing employment outcomes^a^	Results
1	Agarwal et al. (2017)^1^	To assess factors associated with the return to work of head and neck cancer survivors from low‐ and middle‐income countries, such as India	Cross‐sectional survey *n* = 250	24–60 years, Male 87.6%, Female 12.4%, Head and neck cancer	EORTC‐QLQ‐C30, EORTC‐QLQ‐H&N35	Further studies are needed to address the large unmet needs regarding identification and amelioration of barriers to return to work for head and neck cancer survivors in low‐ and middle‐income countries, such as India
2	Bouhnik et al. (2015)^2^	To describe the design and implementation of VICAN (VIe après le CANcer), a national survey on French cancer survivors including multiple domains including employment	Study design report	18–82 years, Mixed gender, Mixed Cancer Cohort	N/A	No results reported as such
3	Cheng et al. (2016)^3^	To explore the symptom burden (cognitive limitation and psychological distress) of employed breast cancer survivors in Hong Kong, and to investigate whether such factors are related to work productivity and QoL	Cross‐sectional survey, *n* = 90	32–60 years, Female 100%, Breast cancer	Hospital Anxiety and Depression Scale, Cognitive Symptoms Checklist 21_,_ Work Limitation Questionnaire 25, EORTC‐QLQ‐C30	The self‐perceived cognitive limitations at work of breast cancer survivors were significantly higher than that of the healthy control group (5.33 vs. 2.60; *p* < 0.05). The cognitive limitations in breast cancer survivors were significantly associated with their QoL (b Z e0.320; p Z 0.032). A negative relationship be‐ tween depression and QoL in breast cancer survivors was also observed in this study
4	Dahl et al. (2014)^4^	To evaluate work status at 3 months after radical prostatectomy in patients with prostate cancer in relation to sociodemographics, urinary incontinence and bother, medical complications health‐related quality of life and surgical methods	Cross‐sectional survey *N* = 264	Mean age 59.2 years, Male 100%, Prostate cancer	Extended Prostate Cancer Index Composite‐50, Short Form‐12	Change of physical health‐related quality of life was the only factor remaining significantly associated with declined work status in the multivariate analysis. Half of the patients had prolonged immediate sick leave. Having physically strenuous work was the strongest predictor for this outcome
5	Dorland et al. (2017)^5^	To identify work functioning trajectories in the year following return to work in cancer patients and examine baseline sociodemographic, health‐related and work‐related variables associated with work functioning trajectories	Longitudinal cohort study *n*‐384	18–65 years, Male 35%, Female 65%, Mixed Cancer Cohort	Work Role Functioning Questionnaire 27, EORTC‐QLQ Cognitive Symptom Checklist Work Dutch Version, Checklist Individual Strength 8_,_ Patient Health Questionnaire 8_,_ International Standard Classification of Occupation −08, Copenhagen Psychosocial Questionnaire_,_ Work Involvement Scale	Cancer patients with persistently high work functioning had less time between diagnosis and return to work and had less often a changed meaning of work, while cancer patients with persistently low work functioning reported more baseline cognitive symptoms compared to cancer patients in the other trajectories
6	Duclos et al. (2014)^6^	To determine total/subtotal colectomy with ileorectal (IRA) or ileosigmoid (ISA) anastomosis is associated with various reported rates of morbidity, function and quality of life in a series of patients undergoing these operations in our institution	Retrospective cohort study *N* = 320	16.8–90.6 years, Male 51%, Female 49%, Colorectal cancer	Short Form‐12 Physical Component Summary, Short Form‐12 Mental Component Summary	Colectomy with IRA or ISA is safe with low postoperative morbidity and mortality. The employment of IRA and inflammatory bowel disease appear to be independent negative factors on function in multivariate analysis.
7	Duijts et al. (2016)^7^	To explore the influence of change in employment status on health‐related quality of life in cancer survivors' long term after diagnosis, and to identify predictors of work continuation in occupationally active survivors	Longitudinal cohort study *N* = 252	Mean age 50.7 years, Male 29.2%, Female 69.8%, Mixed Cancer Cohort	EORTC‐QLQ, Depression 8 Item Scale, Work Ability Index Functional Assessment of Chronic Illness, Therapy Fatigue Experience and Judgement of Work, Job Content Questionnaire	Cancer survivors ‘continuously working’ function better and have a better health and quality of life than those who are not able to work. However, in occupationally active cancer survivors, one should monitor those with low self‐perceived work ability, because they have an increased risk to discontinue their work
8	Duijts et al. (2017)^11^	To explore the effect of earlier identified factors and expectation of being at work on future employment status in cancer survivors and to assess the degree to which these factors specifically concern cancer survivors, a comparison with heart attack survivors was made.	Longitudinal cohort study *N* = 159	16–65 years, Male 39%, Female 61%, Mixed Cancer Cohort	General Health Questionnaire, Centre for Epidemiological Studies Depression 8 Item Scale CASP19	When predicting future employment status in cancer survivors in the UK, general health is the most relevant factor to consider. While expectation of being at work did not show any significant influence in cancer survivors, in heart attack survivors, it should not be disregarded though, when developing interventions to affect their employment status.
9	Eguchi et al. (2017)^12^	To examine workplace factors and perceptions of return to work opportunities for colleagues with cancer‐related symptoms and/or treatment side effects in Japan	Online, cross‐sectional survey *N* = 3710	20–69 years, Male 50%, Female 50%, Mixed Cancer Cohort	Brief Job Stress Questionnaire	Workplace factors and prior experience of working with a cancer survivor may affect a colleagues' perception of RTW opportunities in Japanese workplaces. Consideration of workplace social factors (workplace support and job control), as well as increased openness and awareness of the particular needs of cancer survivors, is therefore essential to facilitate successful RTW in Japan, as elsewhere
10	Ghasempour et al. (2015)^13^	To determine the rate of return to work and its relation to financial distress among Iranian cancer survivors	Descriptive correlational study *n* = 165	All >18 years, Male 61.8%, Female 38.2%, Mixed Cancer Cohort	Return to Work Questionnaire, Financial Stress/Financial Well‐Being Scale	The findings showed that a high percent of Iranian cancer survivors had not returned to their jobs or considerably reduced working hours after treatment completion. Accordingly, due to high levels of financial distress experienced by participants and its relation to return to work, designing rehabilitation programmes to facilitate cancer survivor return to work should be considered
11	Giuliani et al. (2019)^15^	To determine the prevalence of and factors associated with the reduction or complete cessation of employment following treatment in head and neck cancer survivors	Cross‐sectional study *N* = 130	23–65 years, Male 63.5%, Female 36.5%, Head and neck cancer	Work Status Questionnaire, MD Anderson Symptom Inventory Head and Neck (MDASI‐HN), Cancer Survivors Unmet Needs Measure (CaSUN), EuroQol EQ‐5D‐5L	A high proportion of head and neck cancer survivors reduced their work capacity, and many did not return following cancer treatment. Further research is needed to understand the barriers to work return in these survivors and to explore strategies to encourage resumption of employment and employment satisfaction
12	Ho et al. (2018)^16^	To explore the determinants of employment and suboptimal workability and evaluate the association between workability and patient‐reported physical, psychological, and social outcomes	Cross‐sectional study *N* = 327	48–61 years, Female 100%, Breast Cancer	EORTC‐QLQ‐BR23, Multidimensional Fatigue Inventory, Brief Pain Index	Lower employment and reduced workability in breast cancer survivors is common, and reduced workability is associated with higher levels of depression, financial difficulty and physical fatigue, more breast symptoms, and poorer global health status
13	Horsboel et al. (2015)^17^	To examine levels of fatigue, depression and anxiety following diagnosis of a haematological malignancy, to determine the incidence of return to work (RTW) and long‐term sickness absence (LTSA) during 1‐year follow‐up and to examine whether fatigue, depression and anxiety are associated with RTW and LTSA in this group of cancer patients	Cross‐sectional survey *N* = 196	19–59 years, Male 60% Female 40%, Haematological Cancer	Multidimensional Fatigue Inventory—20, Hospital Anxiety and Depression Scale	Half of sickness absent patients returned to work, and only a few of working patients experienced LTSA during follow‐up. Patients reporting high levels of physical fatigue were less likely to RTW. There was a similar tendency for anxiety, whereas we found no association between depression and RTW
14	Hornbrook et al. (2017)^18^	To assess determinants of market and nonmarket employment, job search, volunteering and homemaking among rectal cancer survivors 5 years or longer after diagnosis	Cross‐sectional survey *N* = 563	Mean age 73.1 years, Male 59.1%, Female 40.9%, Rectal Cancer	City of Hope Quality of Life Colorectal Cancer tool, Memorial Sloan Kettering Cancer Centre Bowel Function Index	Higher comorbidity burdens were associated with lower productivity for men and women rectal cancer survivors. Productive survivors were younger and had lower disease stage and age at diagnosis, higher household income and educational attainment, and fewer comorbidity burdens and workplace adjustments than did non‐productive survivors
15	Hou et al. (2022)^19^	To investigate the employment status, employment readiness and other factors affecting the ease or difficulty with which breast cancer patients effect their return to work	Mixed‐method design *N* = 192 (Quantitative) *N* = 41 (Qualitative)	18+ years Female 100% Breast cancer	Patient Health Questionnaire‐9 (PHQ‐9), the Brief Fatigue Inventory (BFI), the Work Ability Index (WAI), and the Lam Assessment of Employment Readiness (LASER)	Forty‐one breast cancer patients had returned to work. Breast cancer patients have a low level of employment readiness. Nurses and other healthcare providers can develop relevant interventions to promote employment readiness and ultimately achieve RTW in this study population.
16	Ibrahim et al. (2017)^20^	To determine the effectiveness of a 12‐week post‐radiation exercise programme in minimizing upper extremity dysfunction in young adults with breast cancer	Pilot randomized control trial *N* = 59	Mean age 39.2 years, Female 100%, Breast cancer	Disability of Arm, Shoulder, and Hand (DASH), the Metabolic Equivalent of Task hours per week (MET‐hours/week), and a post hoc questionnaire on return to work	Although the short‐term targeted exercise programme had no effect on long‐term upper limb function post‐radiation, timing and program specificity may require consideration of tissue healing post‐radiation and surgery type. The majority of participants returned to work, however not returning to pre‐diagnosis work hours
17	Keim‐Malpass et al. (2016)^21^	To examine whether work‐related perceptions are independently associated with QoL, controlling for sociodemographic and cancer‐related factors, among employed breast cancer survivors 18–26 months post‐diagnosis	Cross‐sectional survey *N* = 258	Mean age years, Female 100%, Breast cancer	Functional Assessment of Cancer Therapy‐Breast	Women who reported greater ability to keep up with work responsibilities had higher QoL scores; having more financial worries was associated with lower QoL scores on all domains
18	Kim et al. (2015)^22^	To derive the overall economic burden of cancer in Korea and examine the changing patterns of direct and indirect costs of cancer care, using the previous nationwide studies as references	Data registry analysis *N* = 860	18–84 years, Male 47%, Female 53%, Mixed Cancer Cohort	Survey, Epidemiology and End Results	A comprehensive cancer survivorship policy aimed at lower caregiving cost and higher rate of return to work has become more important than previously considered
19	Koch et al. (2015)^23^	To investigate employment and psychological factors in head and neck cancer survivors with survivorship of ≥2 years	Exploratory survey *N* = 55	Mean age 53.06 years, Male 80%, Female 20%, Head and neck cancer	Miller Behavioural Style Scale, General Perceived Self Efficacy Scale, Patient Health Questionnaire‐9, Horgenheider‐Fragebogen short version	The rate of employed patients dropped from three‐fourths of patients before diagnosis to one‐third at an average of 66.8 months after diagnosis. Current unemployment was associated with harder physical work before cancer diagnosis, surgical treatment and current risky alcohol consumption. Unemployed survivors reported decreased FACT functional and social well‐being and higher PHQ depression scores
20	Lee et al. (2015)^24^	To determine the association of job status and HRQOL with respect to particular situations, such as long‐term cancer survival and the presence of comorbid conditions, as compared with the general population	Data registry analysis *N* = 27,089	20–78 years, Male 43%, Female 57% Mixed Cancer Cohort	EuroQol five‐dimension instrument	There was a significant association between job status and health‐related quality of life in study participants. This study suggests that individuals who are long‐term cancer survivors or have comorbid conditions need particular attention, and a specialized job rehabilitation programme should be developed
21	Leensen et al. (2017)^25^	To investigate return to work rates of cancer patients and to evaluate changes in work‐related quality of life and physical outcomes	Longitudinal prospective intervention study using a one‐group design. *N* = 93	Mean age 47.9 years, Male 10%, Female 90%, Mixed Cancer Cohort	Work Ability Index, Lagerveld 11‐item self‐efficacy scale, Work Limitations Questionnaire, Multidimensional Fatigue Inventory	Return to work rates of cancer patients were high after completion of the multidisciplinary rehabilitation programme. A multidisciplinary rehabilitation programme which combines occupational counselling with a supervised physical exercise programme is likely to result in return to work, reduced fatigue and increased importance of work, work ability and quality of life
22	Magyari et al. (2017)^26^	To evaluate psychological distress and its risk factors among Hodgkin Lymphoma survivors	Cross‐sectional Survey *N* = 163	Mean age 44.82 years, Male 50%, Female 50%, Hodgkins Lymphoma	Hospital Anxiety and Depression Scale, General Health Questionnaire, Sense of Coherence—13 Perceived Stress Scale, Dysfunctional Attitude Scale	Data suggest that employment status and treatment‐related long‐term side effects play a critical role in the health‐related quality of life outcome among Hungarian Hodgkin Lymphoma survivors
23	Moskowitz et al. (2014)^27^	To predict work ability (whether survivors reported lower work ability following cancer) and work sustainability (whether survivors had ever lost or left a job because of cancer, that is work retention) in cancer survivors in the United States	Cross‐sectional survey *N* = 1525	20–74 years, Male 39.4%, Female 61.6%, Mixed Cancer Cohort	Brief Pain Inventory, Distress Thermometer Hospital Anxiety and Depression Scale, Fear of Progression Questionnaire, Short Form Health Survey	Functional limitations and problems at work including poor treatment, discrimination, being passed over for promotion and lack of accommodations were directly related to the ability to work. Problems at work were associated with lower work sustainability
24	Maheu et al. (2021)	To assess the usability and utility of two work‐focused tools to support return to work and maintain work following cancer	Cross‐sectional survey *N* = 28	Mean age 46 years (range 31–65 years), Male 32%, Female 68% Cancer survivors (type unspecified), healthcare service providers, employers, other	Cancer and Work Website, Job Analysis Tool, Return to Work Planner Tool	The study documented the value of the tools and the website to support the RTW process as assessed by several key knowledge user groups. The JAT is considered a helpful procedure to identify job demands in order to guide job accommodations
25	Nekhlyudov et al. (2016)^28^	To examine how insurance coverage, financial status and employment vary for survivors of different cancer types.	Cross‐sectional survey *N* = 615	65 years or lower Male 49.3%, Female 50.7%, Breast, colorectal, lung, prostate, melanoma	Cancer Survivor Supplement of Medical Expenditures Panel Survey	Negative employment and financial implications were most common among those with lung, breast and colorectal cancer, and those diagnosed before age 65
26	Tamminga et al. (2016)^29^	To obtain insight into employment and insurance outcomes of thyroid cancer survivors and to examine the association between not having employment and other factors including quality of life	Cross‐sectional survey *N* = 223	Mean age 49.5 years, Male 49%, Female 51%, Thyroid cancer	EORTC‐QLQ Comorbidity Questionnaire, Hospital Anxiety and Depression Scale, THYCA‐Quality of Life Fatigue Assessment Scale, Short Form‐12	In a multivariate logistic regression analysis, higher age, higher level of fatigue and lower educational level were associated with not having employment. Employment was associated with higher quality of life
27	Tevaarwerk et al. (2015)^30^	To develop a better understanding of how metastatic cancer affects employment is a necessary step towards the development of tools for assisting survivors in this important realm	Cross‐sectional survey *N* = 680	45–65 years, Male 31%, Female 69% Mixed Cancer Cohort	Modified MD Anderson Symptom Inventory	A significant percentage of the metastatic patients remained employed; increased symptom burden was associated with a change to no longer working. Modifiable factors resulting in work interference should be minimized so that patients with metastatic disease may continue working if this is desired. Improvements in symptom control and strategies developed to help address workplace difficulties have promise for improving this aspect of survivorship
28	Ullrich et al. (2018)^31^	To analyse return to work among prostate cancer survivors 12 months after having attended a cancer rehabilitation programme and to identify risk factors for no and late return to work	Longitudinal cohort study *N* = 711	Mean age 57.0, Male 100%, Prostate cancer	Hospital Anxiety and Depression Scale, EORTC‐QLQ‐C30, Effort‐Reward Imbalance at Work Questionnaire, Screening Instrument Work and Occupation Occupational Stress and Coping Inventory	A high proportion of prostate cancer survivors return to work after a cancer rehabilitation programme. However, results indicate the necessity to early identify survivors with low return to work motivation and unfavourable work‐ related perceptions who may benefit from intensified occupational support during cancer rehabilitation
29	van Egmond et al. (2016)^32^	To identify factors and motives associated with (non‐) participation of cancer survivors with job loss in the return to work programme	Cross‐sectional survey *N* = 286	Mean age 49 years, Male 31%, Female 69%, Mixed Cancer Cohort	EORTC‐QLQ, Depression 8 Item Scale, Functional Assessment of Chronic Illness, Therapy Fatigue Work Ability Index, Utrecht Scale of Revalidation and Participation, Readiness to RTW instrument	Being married or living together decreased the likelihood of participation in the RTW programme. Having a temporary employment contract prior to unemployment, a clear intention to return to work and higher scores on a readiness to return to work instrument, that is contemplation scale and prepared for action–self‐evaluative scale, increased the likelihood of participation. Physical and mental problems were leading motives for declining participation
30	van Egmond et al. (2016)^33^	To assess the effectiveness of the programme on duration until sustainable return to work in cancer survivors with job loss	Randomized control trial *N* = 171	Mean age 48.4 years, Male 31%, Female 69%, Mixed Cancer Cohort	EORTC‐QLQ, Functional Assessment of Chronic Illness, Therapy Fatigue Utrecht Scale of Revalidation and Participation	As the tailored return to work programme did not demonstrate a statistically significant effect on duration until sustainable return to work in cancer survivors with job loss, implementation of the programme in its current form is not recommended
31	van Egmond et al. (2016)^34^	To evaluate the likelihood of theory and implementation failure, as well as to evaluate procedures for recruitment, execution and implementation of the tailored return to work programme for cancer survivors with job loss	Single arm trial *N* = 85	Mean age 47.9 years, Male 28%, Female 72%, Mixed Cancer Cohort	No tools	Participants, occupational health care professionals, re‐integration coaches and job‐hunting officers generally had positive experiences with the innovative tailored return to work programme
32	van Maarschalkerweerd P, Rademakers J, Rijken M. (2017)^35^	To explore cancer survivors' level of patient activation, that is their knowledge, skills and confidence for self‐management, and to examine its relations to their participation in paid work and work‐related problems	Cross‐sectional survey *N* = 524	Mean age 18–64 years, Male 51%, Female 49%, Mixed Cancer Cohort	Patient Activation Measure‐ 13 Research and Development General Health Scale	Patient activation was not associated with participation in paid work. Employed cancer survivors with a low level of patient activation experienced more problems working accurately (34% vs. 17%), finishing their work (47% vs. 22%) and concentrating (59% vs. 31%) than those with a higher level of patient activation
33	Yagil et al. (2019)^36^	To identify factors that predict the extent to which healthcare professionals view involvement in the return to work of cancer survivors as part of their role	Cross‐sectional survey *N* = 157	Mean age 47.9, Male 20%, Female 80%, Mixed Cancer Cohort	RTW Role Responsibility Questionnaire	Both belief in the benefits of return to work, and the view that return to work is the team responsibility of healthcare professionals working with cancer survivors, are positively related to viewing return to work as part of the responsibilities of one's personal professional role
34	Yu et al. (2018)^37^	To evaluate the impact of myeloproliferative neoplasms on employment, career potential and work productivity	Cross‐sectional survey *N* = 904	Mean age 54 years, Male 29.4%, Female 70.6% Haematological cancers	Work Productivity and Activity Impairment Specific Health Problem questionnaire	Half of the employed respondents had an employment status change (e.g. leaving a job, medical disability leave, early retirement) because of their disease since the diagnosis. Currently employed respondents reported meaningful impairments in work productivity and activities of daily living that were attributable to their MPNs, and the degree of impairments highlighted the severity of symptom burden
35	Zaman et al. (2016)^38^	To study the effectiveness on return to work of an early tailored work‐related support intervention in patients diagnosed with curative gastrointestinal cancer	Randomized control trial *N* = 88	Mean age 55 years, Male 67%, Female 33%, Gastrointestinal cancers (oesophagus, stomach, liver, pancreas, biliary, small intestine, colon or rectum cancer)	Short Form‐12 Physical Component Summary, Work Limitation Questionnaire, EORTC QLQ‐C30	Patients in the intervention group seem to take fewer days to return to work, albeit not to a statistically significant extent

*Note*: The Cognitive Symptom Checklist—Work in cancer patients (CSC‐W Dutch Version). European Organization for the Research and Treatment Quality of Life Questionnaire (EORTC‐QLQ). European Organization for the Research and Treatment of Breast Cancer Quality of Life Questionnaire (EORTC‐QLQ‐BR23). European Organization for the Research and Treatment of Cancer Quality of Life Questionnaire (EORTC‐QLQ‐C30). European Organization for the Research and Treatment of Head & Neck Cancer Quality of Life Questionnaire (EORTC‐QLQ‐H&N35). European Quality of Life Scale 5D Version (EuroQol 5D). European Quality of Life Scale 5D Version—5 Level (EuroQol EQ‐5D‐5L). Functional Assessment of Cancer Therapy (FACT Fatigue Scale).

^a^ Control, Autonomy, Self‐Realization and Pleasure (CASP19).

Sixteen studies examined work‐related issues using cohorts of mixed cancer types. The remaining 19 studies specifically investigated one type of cancer, for example head and neck,^37^, ^41^ breast,^8^, ^14^, ^38^, ^57^ prostate,^39^, ^56^ haematological,^67^ hepatic (liver),^9^ thyroid^47^ and myoproliferative.^44^ Four studies focused on the employment needs from the perspective of colorectal cancer survivors.^43^, ^59^, ^62^, ^68^ All studies used a variety of tools to assess employment‐related matters for cancer survivors.

### 3.3 Tools used to identify employment‐related factors in cancer survivors

From the 35 included studies, a total of 77 tools were identified that assessed some aspect related to employment (Tables 2 and 3). Only 15 tools were specific to work outcomes such as the Work Limitations Questionnaire. The majority of tools included were those assessing factors potentially influencing work outcomes such as stress (e.g. Perceived Stress Scale) or fatigue (e.g. multidimensional Fatigue Inventory). Of these, the Hospital Anxiety and Depression Scale (HADS) was the most frequently used tool across all cancer types. Most of these tools were originally designed for populations other than cancer (e.g. HADS). This tool was included in six studies for breast, haematological, mixed, prostate and thyroid cancers.^38^, ^44^, ^46^, ^47^, ^56^, ^67^ The second most used measure was a quality‐of‐life scale, The European Organisation for Research and Treatment of Cancer‐Quality of Life Questionnaire (EORTC‐QLQ‐C30). This was included in three studies that focused on breast, colorectal, head and neck, and thyroid cancers.^37^, ^38^, ^56^ The following measurement tools were used in more than one type of cancer: Multidimensional Fatigue^8^, ^65^, ^67^ and Short Form‐12.^39^, ^46^, ^47^, ^62^ All the remaining tools were only used by studies to examine one type of cancer.

**TABLE 3:** Tools identified assessing employment‐related factors in cancer survivors in included studies.

No. (for Monica only, will be deleted later)	Measurement Tools	Breast Cancer (*n* = 5, *t* = 14)	Colorectal Cancer (*n* = 3, *t* = 4)	Haematological Cancer (*n* = 2, *t* = 6)	Head and Neck Cancer (*n* = 3, *t* = 11)	Hepatic (liver) Cancer (*n* = 1, *t* = 1)	Mixed cancer diagnoses (*n* = 19, *t* = 42)	Myoproliferative (*n* = 1, *t* = 1)	Prostate Cancer (*n* = 2, *t* = 6)	Thyroid Cancer (*n* = 1, *t* = 5)
1	Attitudes‐Social Influence Self Efficacy Questionnaire						√			
2	Brief Fatigue Inventory	√								
3	Brief Pain Index	√								
4	Brief Pain Inventory						√			
5	Brief Job Stress Questionnaire						√			
6	Cancer Survivors Unmet Needs Measure				√					
8	CASP19						√			
9	Centre for Epidemiological Studies Depression 8 Item Scale						√			
10	Checklist Individual Strength 8 (4)						√			
11	City of Hope Quality of Life Colorectal Cancer tool		√							
12	Cognitive Symptoms Checklist 21	√								
13	Comorbidity Questionnaire									√
14	Copenhagen Psychosocial Questionnaire						√			
15	CSC‐W Dutch Version						√			
16	Distress Thermometer						√			
17	Disability of Arm, Shoulder and Hand	√								
18	Dutch Occupational Impact on Sleep Questionnaire						√			
19	Dysfunctional Attitude Scale			√						
20	Effort‐Reward Imbalance at Work Questionnaire								√	
21	EORTC‐QLQ						√			√
22	EORTC‐QLQ‐BR23	√								
23	EORTC‐QLQ‐C30	√			√				√	
24	EORTC‐QLQ‐H&N35				√					
25	EuroQol 5D						√			
26	EuroQol EQ‐5D‐5L				√					
27	Experience and Judgement of Work						√			
28	Extended Prostate Cancer Index Composite‐50								√	
29	FACT Fatigue Scale						√			
30	Fatigue Assessment Scale									√
31	Fear of Progression Questionnaire						√			
32	Financial Stress/Financial Well‐Being Scale						√			
33	Functional Assessment of Cancer Therapy				√	√				
34	Functional Assessment of Cancer Therapy‐Breast	√								
35	Functional Assessment of Chronic Illness Therapy Fatigue						√			
36	General Health Questionnaire						√			
37	General Perceived Self Efficacy Scale				√					
38	General Health Questionnaire			√						
39	Horgenheider‐Fragebogen short version				√					
40	Hospital Anxiety and Depression Scale	√		√			√		√	√
41	International Standard Classification of Occupation ‐ 08						√			
42	Job Analysis (Cancer and Work)						√			
43	Job Content Questionnaire						√			
44	Lam Assessment of Employment Readiness	√								
45	Lagerveld 11‐item Self efficacy Scale						√			
46	Memorial Sloan Kettering Cancer Centre Bowel Function Index		√							
47	Metabolic Equivalent of Task Hours per week	√								
48	MD Anderson Symptom Inventory Head and Neck									
49	Miller Behavioural Style Scale									
50	Multidimensional Fatigue Inventory	√					√			
51	Multidimensional Fatigue Inventory—20			√						
52	Modified MD Anderson Symptom Inventory						√			
53	Occupational Stress and Coping Inventory								√	
54	Patient Activation Measure‐ 13						√			
55	Patient Health Questionnaire 8						√			
56	Patient Health Questionnaire‐9	√			√					
57	Perceived Stress Scale			√						
58	Problem Questionnaire							√		
59	Quality of Working Life Questionnaire for Cancer Survivors						√			
60	Readiness to RTW Instrument						√			
61	Research and Development General Health Scale						√			
62	Return to Work Questionnaire (Nillson 2013)						√			
63	Return To Work Role Responsibility Questionnaire						√			
64	Sense of Coherence—13			√						
65	Screening Instrument Work and Occupation								√	
66	Short Form‐12		√						√	√
67	Short Form Health Survey						√			
68	Survey, Epidemiology and End Results						√			
69	THYCA‐QoL									√
70	Utrecht Coping List						√			
71	Utrecht Scale of Revalidation and Participation						√			
72	Work Ability Index	√					√			
73	Work Involvement Scale						√			
74	Work Limitation Questionnaire Short Version		√				√			
75	Work Limitation Questionnaire	√								
76	Work Productivity and Activity Impairment Specific Health							√		
77	Work Role Functioning Questionnaire						√			
78	Work Status Questionnaire				√					

Abbreviations: *N*, number of studies; T, number of tools.

### 3.4 Characteristics of tools assessing employment‐related factors in colorectal cancer

Four of the 77 tools were used in the three studies that involved a focus on colorectal cancer: Short Form‐12, City of Hope Quality of Life Colorectal Cancer tool, Memorial Sloan Kettering Cancer Centre Bowel Function Index and the Work Limitations Questionnaire (WLQ)^43^, ^59^, ^62^, ^68^ (Table 4). All tools were self‐reported outcome measures, featuring between 12 and 47 items. Only one tool, the WLQ, assessed a work‐related outcome. The WLQ has previously been demonstrated to be valid populations with chronic health conditions.^69^ Similarly, the SF‐12 was designed for assessing quality of life for any health condition and the general population. All these tools are standardized and have had some level of psychometric testing undertaken.

**TABLE 4:** Characteristics of tools assessing employment‐related factors in colorectal cancer.

Tool name/area	Tool format/purpose	Number of items	Administered by	Target population for which the tool was designed	Time required to administer tool
City of Hope Quality of Life Colorectal Cancer Tool	Questionnaire: an adult patient self‐report instrument designed to assess quality of life	90 items (47 in part one, 43 in part two)	Self‐report	Colorectal Cancer	Not provided
Memorial Sloan Kettering Cancer Centre Bowel Function Index	Questionnaire: to assess the bowel function of patients with rectal cancer undergoing surgery	18 items	Self‐report	Colorectal Cancer	Not provided
Short Form‐12	Questionnaire: generic assessment of health‐related quality of life (HR QOL) from the client/patient's perspective	12 items	Self‐report	All health conditions	5–10 min
Work Limitations Questionnaire	Questionnaire: to measure the degree to which health problems interfere with specific aspects of job performance and the productivity impact of these work limitations	25 items (original) and 8 items (short version)	Self‐report	MSD	5–10 min

Abbreviation: MSD, musculoskeletal disorders.

The four tools that were relevant to colorectal cancer were then mapped against the Mehnert model (Table 5). As seen in Table 5, none of the identified tools considered all factors/variables or outcomes of Mehnert model. Three of the tools, City of Hope Quality of Life, Short Form‐12 and the Memorial Sloan Kettering Cancer Centre Bowel Function Index included a total of three features of Mehnert's model. These tools were not consistent in the features included, with only one category of mediator variable (Impairments and health status of the individual) that was similar across all. Of interest, none of the tools included features about disease specific factors (Table 5, column 1), factors that may motivate the individual to work (e.g. work satisfaction; meaning of work, Table 5, column 6), the influence of work‐related interventions (Table 5, column 8) or potential economic outcomes (Table 5, column 11).

**TABLE 5:** Mapping of CRC tools against the Mehnert framework.

Tool	1	2	3	4	5	6	7	8	9	10	11
City of Hope Quality of Life Colorectal Cancer Tool				√	√					√	
Memorial Sloan Kettering Cancer Centre Bowel Function Index		√	√	√							
Short Form‐12				√	√					√	
Work Limitation Questionnaire							√		√		

*Note*: 
Disease specific factors (cancer stage; time since diagnosis; disease phase; clinical characteristics; cancer site). Treatment‐related factors (surgery; chemotherapy; radiation; medication; treatment intention; endocrine therapy; multimodal treatment). Demographic factors (age; gender; education; income; ethnicity; marital status; social status). Impairments and health status (bodily impairments; cognitive impairments; comorbid diseases). Psychosocial factors (psychological symptoms; psychological comorbidity; social skills/support). Motivational factors (intention to work; work satisfaction; meaning of work). Work‐related factors (type of work, work demands, work sector; level of responsibility; work environment; employer accommodation; relationship with co‐workers and managers). Work‐related interventions (counselling, vocational training services; job replacement services; job search assistance; rehabilitation services; continuity of care). Work‐related outcomes (employment, return to work, continuity of work; work ability; sick leave, length of absence from work; work changes; work productivity; burden at work, perceived job strain; work disabilities; job satisfaction; income levels, earnings retirement; unemployment, job loss, to quit working; reemployment, time of returning to work; reduction in work hours; temporary work weekly work hours; career change, promotion). Psychosocial outcomes (QoL, psychological well‐being, body changes, self‐evaluation, life interference social participation; social reintegration and work gratification). Economic outcomes (economic costs of, for example decline in work productivity, time of returning to work, early retirement, sick leave and absence from work).

## 4 DISCUSSION

In this scoping review, 11,704 journal articles were screened with 35 articles selected to examine the current evidence relating to readiness for return to work or remain at work for people with cancer including colorectal cancer. Most of the published research identified was focussed on cancer in general. It is evident that there are only a limited number of studies specifically focussed on colorectal cancer that utilize tools to identify employment concerns.

The 35 studies included in this review generally demonstrated that there are numerous barriers to returning to work after a diagnosis of, or treatment for cancer. Those that do return with a higher degree of comorbidity were shown to have greater difficulty re‐integrating into the work environment.^14^ Several studies demonstrated that reduced capacity to return to, or engage fully in work was associated with poorer quality of life outcomes.^8^, ^9^, ^10^

### 4.1 Aim 1: to identify available measures related to employment readiness in cancer survivors especially colorectal cancer

Several tools were identified that assess employment readiness in cancer survivors. While these were mostly well‐established and standardized tools, none addressed return‐to‐work or remain‐at‐work for colorectal cancer survivors specifically but rather perceived barriers to work. Indeed, all tools (Tables 2 and 3) were designed for use in patients with health conditions other than cancer. For example, tools designed to assess pain in those with musculoskeletal problems (Brief Pain Inventory/Index), were used with breast cancer clients.^8^ Most studies that used a measure of pain also assessed other factors (e.g. quality of life) in recognition that employment readiness is a multifactorial outcome. This suggests that in the absence of a comprehensive tool for the cancer population, pain should not be considered in isolation of other factors.

This review identified four tools that have been used to explore return‐to‐work among colorectal cancer survivors. All are self‐report tools, as opposed to clinician‐rated tools, of which two were designed to assess global quality of life. The first tool is the Short Form‐12 which is a generic assessment of health‐related quality of life from the patient's perspective while the City of Hope Quality of Life Colorectal Cancer Tool includes factors specific to colorectal cancer—for example, the presence of a temporary or permanent ostomy. The City of Hope tool consists of two parts. The first part includes 47 items that relate to the patient's sociodemographic characteristics including work‐related items, health insurance, sexual activity, psychological support, clothing, diet and daily ostomy care. The second component includes 43 quality of life items that use 10‐point Likert scales for recording responses. The quality‐of‐life component is further divided into four domains or subscales: physical well‐being (items 1–11), psychological well‐being (items 12–24), social well‐being (items 25–36) and spiritual well‐being (items 37–43). The psychometric properties of this tool have not been confirmed.^57^ The one study that used the City of Hope tool identified several demographic (younger age, higher household income, educational attainment), disease‐related (lower disease stage and age at diagnosis and fewer comorbidity burdens) and workplace (workplace adjustments) factors associated with being employed compared with not being employed.^43^ This suggests the need to evaluate a range of factors. However, it may be possible there are a few unique factors that are essential rather than the 89 items.

In contrast, the Short Form‐12 is a shortened version of Short Form‐36, which itself evolved from the Medical Outcomes Study for use among people with any health condition. The Short Form‐12 included eight domains: (1) limitations in physical activities because of health problems; (2) limitations in social activities because of physical or emotional problems; (3) limitations in usual role activities because of physical health problems; (4) bodily pain; (5) general mental health; (6) limitations in usual role activities because of emotional problems; (7) vitality and (8) general health perceptions. While quality of life is relevant when considering employment readiness, neither of the above tools individually captures all features important to assessing the colorectal cancer survivor's ability to return to work. The City of Hope tool thoroughly addresses one issue, that is the presence of colostomy bags, which is an issue that presents unique challenges and barriers to work performance. This has indeed been shown to be one of the primary concerns of colorectal cancer survivors due to the changes they cause in bodily and social function.^19^ However, this tool lacks a more nuanced examination of overall work function or dysfunction and all questions are specific to the presence of an ostomy, which is not necessarily an outcome for all colorectal cancer survivors. Conversely, the Short Form‐12 provides only a generic assessment of health, which remains applicable to all colorectal cancer survivors; however, it lacks depth in treatment and disease specific effects and outcomes.

The final two tools used to examine work outcomes in colorectal cancer survivors were the Memorial Sloan Kettering Cancer Centre Bowel Function Index and the Work Limitations Questionnaire. The Memorial Sloan Kettering index contains 18 questions designed to assess bowel function after sphincter‐preserving surgery for rectal cancer. It details information about a person's diet, urgency and frequency post‐surgery, but there are no questions related to employment. On the contrary, the Work Limitations Questionnaire was designed to measure the degree to which health conditions interfere with specific components of job performance and the productivity impact of these work limitations. It is a generic tool used for chronic health conditions. Of the two tools above, one measures post‐colorectal cancer surgery related issues, the other measures work‐related issues which are both important components to be included in an employment readiness related tool. It is possible that a combination of the two tools could capture a comprehensive understanding of colorectal cancer survivors' work‐related issues. Return‐to‐work is a complex process and each of the four tools discussed above capture various elements of the process. A truly comprehensive tool would require an approach examining each element for a nuanced understanding of an individual colorectal cancer survivor's readiness for work.

### 4.2 Aim 2: chart the relevant factors of employment against Mehnert's model

#### 4.2.1 Main findings

For this review, the model used to chart the factors relevant for employment in cancer survivors was that by Mehnert.^35^ A unique feature of this model is that it not only considers factors relevant to work but a range of employment outcomes such as continuity of work; work ability; sick leave, length of absence from work; work changes; work productivity; and burden at work. Of the four tools specific to the assessment of employment in colorectal cancer survivors in this review, none mapped against all the features identified in Mehnert's model.

The WLQ was the tool that included two items addressing work‐related factors and outcomes. Identifying work‐related outcomes is an important consideration in the selection of any tool. These outcomes consist of a wide range of variables such as continuity of work, employment and return to work process, work ability, absence from work and career changes. This also incorporates dimensions of quality of life, such as social reintegration and psychological well‐being as well as—though less frequently—economic variables such as costs of decline in work productivity. The WLQ systematically measures the degree to which health problems interfere with specific aspects of job performance and the productivity impact of these work limitations. However, it lacks inclusion of some of the cancer‐specific factors, both disease and treatment related, that are relevant when considering the return‐to‐work process. These include some health and disease‐related factors both pre‐ and post‐diagnosis, such as stage of cancer, cancer site and physical fitness level pre‐diagnosis. Also, absent were treatment‐related factors including chemotherapy agent, and associated nausea, vomiting and cognitive dysfunction, radiotherapy medium, hormonal therapy, postoperative infection rate, frequency of follow‐up appointments and cancer‐related fatigue. These omissions may therefore limit the appropriateness of the WLQ for use among colorectal cancer survivors.

This scoping review applied an approach of assessing the outcome criteria examined by tools used in the setting of returning and remaining at work after a cancer diagnosis. Namely, it used the Mehnert framework as a means by which to interrogate the applicability of a number of tools to the return‐to‐work process for colorectal cancer. To ensure that tools remain contemporary, an updated assessment tool would recognize a greater number of factors identified in the Mehnert framework. Factors such as sociodemographic factors; health and disease‐related factors; treatment‐related factors; psychological factors and work‐related factors have emerged as important to consider when returning to work.^35^

As generic tools lack the necessary focus on the work‐limiting effects of symptoms specific to colorectal cancer, the development of a new tool will require the input of colorectal cancer survivors. This input will ideally help elucidate which symptoms of their cancer and its treatment affect their ability to return‐to‐work. This review has found that only a minority of generic tools thoroughly investigated effects on working life. It will therefore be important to have the input of survivors to determine which aspects of their working life changed due to their cancer and its treatment.

## 5 STUDY LIMITATIONS

The strength of this review lies in the identification of few colorectal cancer‐specific studies. This enabled the planning of the search strategy to include all cancer types so that broader lessens could be learned that can inform future tool development for colorectal cancer survivors. This review only searched the abstract, title, subject headings and keywords in several databases, which may not have yielded a complete pool of relevant articles. Articles published in other languages (not in English) were also excluded. The omission of grey literature meant that websites and tools developed by cancer agencies were not included, as while they may yield potentially useful information, they have not been subject to scientific scrutiny.

## 6 CLINICAL IMPLICATIONS/CONCLUSION

Currently, the reporting of return‐to‐work issues and employability following colorectal cancer specifically is limited, with no tools available to comprehensively address one of the most common cancers in the world. With improving medical treatment and cancer survivorships, employment and return to work becomes increasingly important for all cancer survivors in their quality of life. A measurement tool for return‐to‐work in colorectal cancer survivors will help healthcare professionals and policymakers to obtain a more detailed and insightful understanding of people's well‐being beyond the medical diagnosis of cancer. Such a tool would likely incorporate fields examined by existing tools to ensure that physiological and employment‐related components were assessed holistically. With quantifiable data, it is possible to develop a pathway to improve post‐cancer‐treatment and care, with the aim of maximizing recovery and return of function.

## AUTHOR CONTRIBUTIONS

**Mingshuang Ding:** Conceptualization (lead); data curation (lead); formal analysis (lead); investigation (lead); methodology (lead); project administration (lead); writing – original draft (lead); writing – review and editing (lead). **Elise Gane:** Investigation (equal); methodology (equal); supervision (equal); writing – original draft (equal); writing – review and editing (equal). **Harry Wiffen:** Writing – original draft (equal); writing – review and editing (equal). **Venerina Johnston:** Conceptualization (equal); formal analysis (equal); methodology (equal); supervision (equal); writing – review and editing (equal).

## CONFLICT OF INTEREST STATEMENT

The authors declare no conflicts of interest.

## Data Availability

Not applicable.
